# High resolution MRI for quantitative assessment of inferior alveolar nerve impairment in course of mandible fractures: an imaging feasibility study

**DOI:** 10.1038/s41598-020-68501-5

**Published:** 2020-07-14

**Authors:** Egon Burian, Nico Sollmann, Lucas M. Ritschl, Benjamin Palla, Lisa Maier, Claus Zimmer, Florian Probst, Andreas Fichter, Michael Miloro, Monika Probst

**Affiliations:** 1 Department of Diagnostic and Interventional Neuroradiology, Medical School Munich, Klinikum Rechts der Isar, Technische Universität München, Ismaninger Str. 22, 81765 Munich, Germany; 2 Department of Oral and Maxillofacial Surgery and Facial Plastic Surgery, Klinikum Rechts Der Isar, Technische Universität München, Munich, Germany; 3 Department of Oral and Maxillofacial Surgery, University of Illinois at Chicago, Chicago, IL USA; 4 Department of Oral and Maxillofacial Surgery and Facial Plastic Surgery, University Hospital, LMU Munich, Munich, Germany

**Keywords:** Anatomy, Medical research

## Abstract

The purpose of this study was to evaluate a magnetic resonance imaging (MRI) protocol for direct visualization of the inferior alveolar nerve in the setting of mandibular fractures. Fifteen patients suffering from unilateral mandible fractures involving the inferior alveolar nerve (15 affected IAN and 15 unaffected IAN from contralateral side) were examined on a 3 T scanner (Elition, Philips Healthcare, Best, the Netherlands) and compared with 15 healthy volunteers (30 IAN in total). The sequence protocol consisted of a 3D STIR, 3D DESS and 3D T1 FFE sequence. Apparent nerve-muscle contrast-to-noise ratio (aNMCNR), apparent signal-to-noise ratio (aSNR), nerve diameter and fracture dislocation were evaluated by two radiologists and correlated with nerve impairment. Furthermore, dislocation as depicted by MRI was compared to computed tomography (CT) images. Patients with clinically evident nerve impairment showed a significant increase of aNMCNR, aSNR and nerve diameter compared to healthy controls and to the contralateral side (p < 0.05). Furthermore, the T1 FFE sequence allowed dislocation depiction comparable to CT. This prospective study provides a rapid imaging protocol using the 3D STIR and 3D T1 FFE sequence that can directly assess both mandible fractures and IAN damage. In patients with hypoesthesia following mandibular fractures, increased aNMCNR, aSNR and nerve diameter on MRI imaging may help identify patients with a risk of prolonged or permanent hypoesthesia at an early time.

## Introduction

In the setting of acute mandibular fractures, the gold standard examination protocol is clinical testing followed by projectional radiography, computed tomography (CT) or cone beam computed tomography (CBCT)^1^. Depending on the location and degree of the fracture, different treatment options may be preferred^2–4^. Although the imaging techniques above can reliably assess bony structures, the involvement of soft tissues cannot be accurately visualized. Mandible fractures involving the IAN-bearing areas of the mandible are commonly associated with insult to the inferior alveolar nerve (IAN) with subsequent neurosensory disturbance^5^.

X-rays, CT, and CBCT imaging can only visualize the bony cortical walls surrounding the mandibular canal^6^. In addition, these radiation-based imaging techniques lack the ability to directly visualize neural structures. For instance, CT scans can display cortical disruption of the bony IAN canal, but this provides no information regarding the fascicular continuity of the IAN, which may be severed or unaffected. For patients with an IAN injury that would benefit from microneurosurgical intervention and repair with an interpositional graft, this is a major shortcoming^7,8^.

Besides trauma, the IAN is at potential risk of injury in wisdom tooth removal, implant placement and orthognathic surgery as well. All of these injuries vary in nature, but the signs and symptoms following nerve damage remain rather uniform, and differ primarily in degree. This classification of nerve injuries was first described by Seddon in 1943^9^, and later revised by Sunderland in 1951^10^. Zuniga et al. previously tried to correlate this histological classification with 3 T MRI imaging parameters^11^. However, the required resolution to visualize the continuity loss of endo- and perineurium was not achieved with a 3 T MRI in that study.

Despite such progress, the “gold standard” for visualizing nerve injuries may still be surgical exposure. Important roles also exist for clinical nerve testing, traditionally including two-point discrimination, brush stroke direction, contact detection, thermal, pin-prick nociception^12^. These methods rely on patient cooperation however, and harbor a degree of subjectivity. Electroneurography or somatosensory evoked potential technics also have their own capabilities and limitations^12–14^. High resolution MR imaging offers an objective means of evaluating neurovascular structures^15^. In cases of nerve damage, preoperative MR imaging could help stratify risks of permanent neurosensory deficits, thus providing an indication for concurrent neurosurgical intervention at the time of fracture repair.

The primary goal of the present study is to evaluate the applicability of a protocol consisting of a 3D STIR, 3D DESS and 3D T1 FFE sequence for rapid imaging assessment of mandible fractures affecting the IAN in the emergency room setting. We also correlate imaging parameters and clinical symptoms in a first step towards gold standard, preoperative exam recommendations.

## Methods and material

### Study design

This prospective MRI study was conducted at the Department of Diagnostic and Interventional Neuroradiology. Fifteen patients suffering from unilateral mandibular angle fractures due to motor vehicle accidents or direct blow from altercations were enrolled (13 male, 2 female). The mean age was 26 ± 10 years (range 16–57 years). Fifteen IAN from the fracture side were compared to fifteen IAN of the contralateral side, which served as the first control. Additionally, 15 healthy age-matched volunteers (8 male, 7 female, mean age: 26 ± 2 years, range 23–31 years) were selected as a second control group (30 IAN). The inclusion criterion was the availability of an existing CBCT or CT (14 CT and 1 CBCT). All volunteers were clinically asymptomatic and had no history or signs of nerve injury. Exclusion criteria were recent oral surgical procedures, a history of cranio-maxillofacial syndromes or diseases and standard contraindications for MRI (e.g. implanted pacemaker). The trial was performed in accordance with ethical guidelines and received institutional review board approval of the Technical University of Munich (approval number 432/18). Written informed consent was given by all subjects.

### Multi-detector computed tomography (MDCT) and cone beam computed tomography

All patients were examined with the same 64-row MDCT scanner (Somatom Sensation Cardiac 64; Siemens Medical Solutions, Erlangen, Germany). MDCT was performed with a standard protocol with the following scanning parameters: 120 kVp tube voltage, adapted tube load of averaged 150 mAs and minimum collimation (0.6 mm).

The CBCT was conducted in a private practice and the scanning protocol was not available. However, the diagnostic accuracy of CBCT and the comparability to CT with regard to dimension stability and precision was outlined before^16,17^.

### MR imaging

All subjects underwent MRI on a 3 T system (Elition, Philips Healthcare, Best, the Netherlands) using a 16-channel head-neck-spine coil, which was placed on top of the head. Subjects were positioned head-first in a supine position^18^. Sequence specifications for the 3D STIR, the 3D DESS and the 3D T1 FFE sequence are listed in Table 1^18^.

**Table 1:** Parameters for the dedicated STIR, DESS and 3D T1 FFE sequences of the inferior alveolar nerve.

**3D STIR**	
Acquisition time	06:03 min
FOV	200 mm
Matrix	308 × 308
Acq voxel	0.65 × 0.65 × 1 mm^3^
Number of signal averages	1
TR	2,300 ms
TE	184 ms
IR	250 ms
Gap	− 0.5 mm
Slice oversample factor	1.5
CS-SENSE	Yes
reduction	5
WFS (pix)/bandwidth (Hz)	1766/246
**3D DESS**	
Acquisition time	05:39 min
FOV	200 mm
Matrix	364 × 308
Acq VOXEL	0.55 × 0.65 × 1 mm^3^
Number of signal averages	1
TR	12 ms
TE1	4.2 ms
TE2	7.7 ms
Gap	− 0.5 mm
WFS (pix)/bandwidth (Hz)	0.607/715
**3D T1 FFE**	
Acquisition time	05:31 min
FOV	180 mm
Matrix	420 × 419
Acq voxel	0.43 × 0.43 × 0.5 mm^3^
Number of signal averages	1
TR	10 ms
TE	1.75 ms
Gap	− 0.25 mm
CS-SENSE	Yes
reduction	2.3
WFS (pix)/bandwidth (Hz)	1503/289

Total scan time was 17:13 min.

#### MR-morphologic assessment

In all included patients the anatomical course of the neurovascular bundle was identified first at each side of the mandible. Then the fracture course was visualized in the T1 FFE “black STIR and 3D DESS sequence. For better visualization of the small sized soft and hard tissue entities coronal and axial planes were used.

### Image analysis

For image analysis, the acquired DICOM datasets were reconstructed in a multiplanar reconstruction (MPR) in an axial and coronal plane, for the purpose of qualitative image analysis. The reconstructed plane was parallel to the fracture dislocation and orthogonal to the course of the IAN. Two board-certified radiologists performed the qualitative and quantitative assessments. Imaging parameters included metric measurements in defined regions of interest (ROI) and computational values like apparent signal-to-noise ratios (aSNR) and apparent nerve-muscle contrast-to-noise ratios (aNMCNR). (see *quantitative image analysis/SNR calculation).*

### Qualitative image analysis

#### Overall image quality assessment

Technical image quality was assessed using a 5-point Likert rating scale (5 = excellent, no restrictions for clinical use; 4 = very good, containing no substantial adverse effect for clinical use; 3 = average, borderline clinical use due to the image quality; 2 = poor, substantial adverse effect for clinical use; 1 = very poor, not suitable for clinical use). Technical image quality was rated with regard to diagnostic confidence, background noise, resolution, artifacts. For 3D STIR and 3D DESS sequences the quality of direct visualization of nerve edema or nerve disruption was crucial for excellent diagnostic confidence while for the 3D T1 FFE sequence fracture detection was the most important imaging criterion.

### Quantitative image analysis

#### Nerve diameter measurements

The nerve diameter of the IAN in all patients was determined using the STIR images in axial reconstructions along their proximo-distal pathways. The IAN diameters were measured in a location approximately 1 cm proximal to the fracture line, and distally within the anterior mandibular body region approximately 1 cm proximal of the detected fracture line. Furthermore, the nerve diameter of the contralateral side was measured in the mandibular body at the corresponding level to the detected fracture line. In the volunteer control group, the IAN diameter was measured for each side at the corresponding level of the fracture course in the patient group.

#### Dislocation comparison of 3D T1 FFE sequence and CT

The fracture dislocation was assessed in the 3D T1 FFE sequence as well as in the CT at maximal separation. For this purpose, the distance between the cortical edge of the proximal and distal segments was used for each patient.

#### SNR and aNMCNR calculation

Signal intensity (SI) of neural structures was measured by drawing a ROI within the course of the IAN. Due to the small size of the measured anatomic structures the ROI had be placed manually in each case. To avoid including posttraumatic edema or hematoma formations to the nerve signal measurements the ROI was placed approximately 1 cm proximal and 1 cm distal to the identified fracture line.

To define the nerve-muscle contrast of the IAN a ROI was placed in the ipsilateral masseter muscle. An area of the Masseter muscle was identified which comprised no vascular structures or posttraumatic, intramuscular edema which would artificially increase the measured signal. The ROIs had a diameter of 3 cm approximately.

The corresponding values were extracted and inserted in two formulas published by Klupp et al.^19,20^:

$${\text{aSNR }}\left( {\text{apparent signal-to-noise ratio}} \right): {\text{SI}}_{{{\text{nerve}}}} /{\text{SD}}_{{{\text{nerve}}}}$$

$${\text{aNMCNR }}\left( {{\text{apparent nerve}} - {\text{muscle contrast-to-noise ratio}}} \right):\left( {{\text{SI}}_{{{\text{nerve}}}} {-}{\text{ SI}}_{{{\text{muscle}}}} } \right)/{\text{SD}}_{{{\text{nerve}}}}$$

#### Clinical neurosensory testing

Clinical neurosensory testing was performed in the course of hospitalization by using two-point discrimination, pin-prick nociception and contact testing^12^. After clinical examination, the patients were classified as having neurosensory impairments if deficits were elicited in any modality, or as experiencing no symptoms if normal testing results.

We have performed 3 neurosensory exams which included all 3 levels of neurosensory testing; (1) 2-point discrimination (Level A), (2) contact testing (Level B), (3) pin-prick (Level C). Similar to Zuniga et al., patients were considered “normal” if no deficits in Levels A–C were identified (2-point discrimination less than 6.5 mm, positive contact identification, and positive pin-prick identification)^12^. Patients were considered abnormal if any deficits were detected in Levels A-C.

### Statistical analysis

The statistical analyses were performed with SPSS (Version 25, SPSS Inc., Chicago, IL, USA). The presented figures were generated using Graphpad Prism Version 8 (version 8.0, GraphPad Software Inc., La Jolla, CA, USA). Computational values were compared between different sequences as well as between proximal and distal segments of the IAN, and between the fracture side and the contralateral side using the nonparametric Mann–Whitney-U test. To assess the reliability of the acquired results, intraclass correlation coefficient (ICC) was calculated. The Mann–Whitney-U test was based on a two-tailed test with a significance level of p < 0.05.

### Ethical approval

The institutional ethics committee approved the study design.

### Informed consent

Informed consent was obtained from all individual participants included in the study.

## Results

### MR-morphologic assessment

Fifteen IANs involved in mandible fractures were included in this study. Serving as controls, 15 IANs of the contralateral side were included, as well as a second control of 30 IANs of healthy, age-matched volunteers. Using the 3D T1 FFE sequence and the 3D STIR sequence, the fracture course and potential IAN involvement could be visualized. The 3D T1 FFE sequence showed precise hard tissue visualization with high spatial resolution. The 3D STIR sequence allowed for edema detection within the IAN. The 3D DESS sequence provided reliable depiction of nerve topography. In contrast to MRI, conventional dental imaging (CT and CBCT) only allowed for the depiction of cortical boundaries within the mandible. Whereas, the STIR sequence allowed for qualitative discrimination of the nerve.

### Quantitative image analysis

#### Image quality assessment

The fracture site and course could be detected with high reliability in each patient. The image quality was “very good” when assessed in the 3D T1 FFE sequence (4.2 out of 5 points on the Likert scale), the 3D STIR sequence (4 out of 5 points) and the 3D DESS sequence (3.9 out of 5 points). The comparison of the two different readers showed “good” to “very good” agreement for the 3D T1 FFE sequence (ICC = 0.812), the 3D STIR (ICC = 0.865) and the 3D DESS (ICC = 0.792).

#### Dislocation comparison of 3D T1 FFE sequence and CT

**Figure 1:**
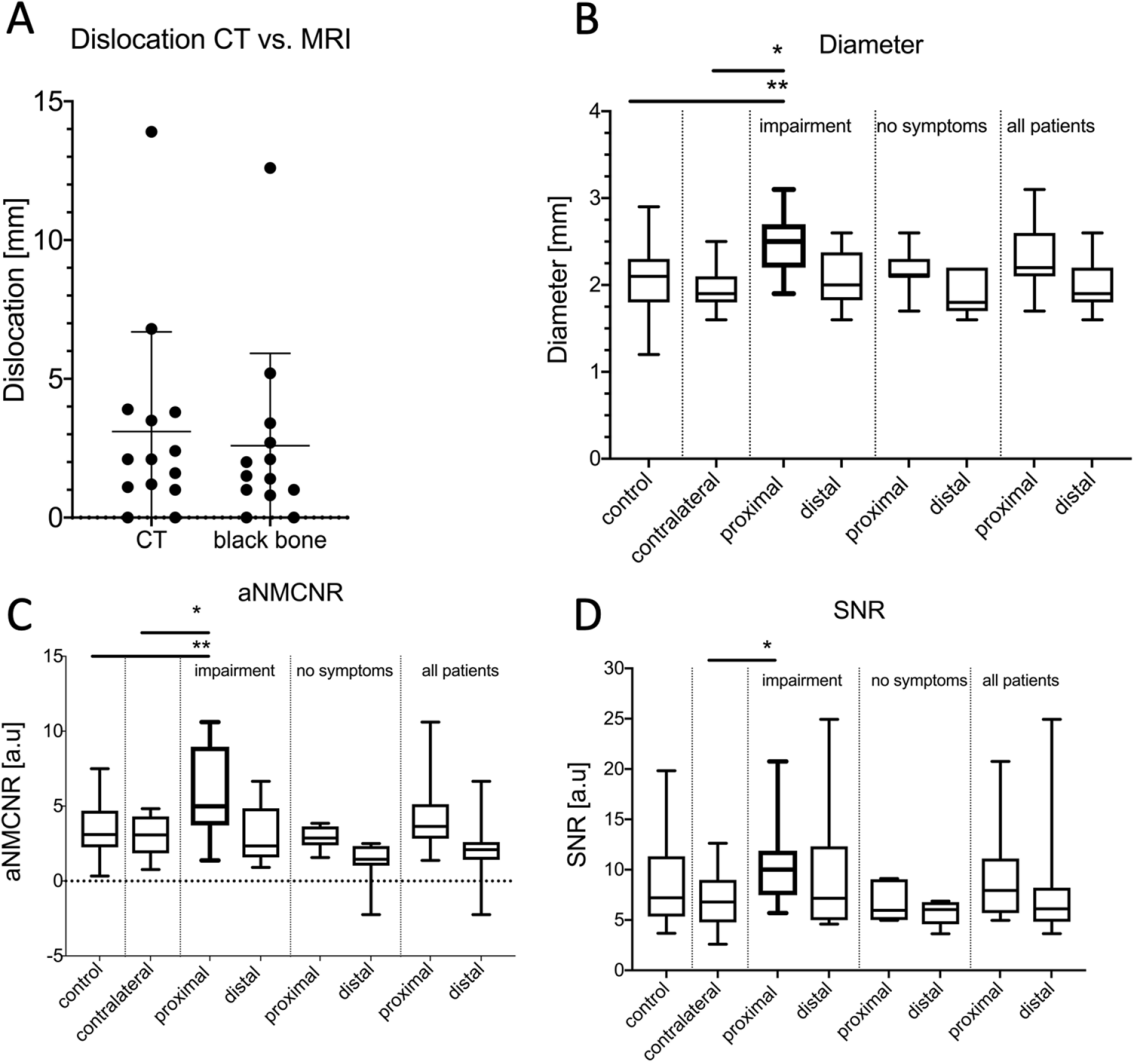
This figure shows the acquired measurements for fracture dislocation in CT and 3D T1 FFE “bone sequence” (**A**). Nerve diameters showed a significant increase proximal of the fracture location (**B**). Further, it could be revealed that aNMCNR (**C**) and aSNR (**D**) were significantly higher at the fractured site compared to the healthy controls and to the contralateral side. (**B**) *p = 0.004, **p = 0.005, (**C**) *p = 0.007, **p = 0.021, (**D**) *p = 0.040.

The dislocation measurements at the punctum maximum of posttraumatic cortical deviation yielded comparable results for the CT and the 3D T1 FFE sequence (3.1 vs. 2.6 mm, p = 0.707). However, a tendency to underestimate the dislocation extent was apparent in the 3D T1 TFE sequence (Fig. 1A). A high ICC was given between the two readers (ICC = 0.999, p < 0.001).

#### Nerve diameter measurements

A significantly enlarged nerve diameter could be detected proximal to the fracture course in patients suffering from neurosensory impairments compared to the contralateral side (2.5 vs. 2.0 mm, p = 0.004) and compared to healthy controls (2.5 vs. 2.1 mm, p = 0.005). However, the distal location had no significant differences compared to the contralateral side (2.1 vs. 2.0 mm, p = 0.625) or to healthy volunteers (2.1 vs. 2.0 mm, p = 0.712). A high intra-observer agreement existed for measurements at the proximal (ICC = 0.959, p < 0.001) and distal locations (ICC = 0.979, p < 0.001). In patients without neurosensory deficits, no significant difference in nerve diameter alterations could be detected (p > 0.05) (Fig. 1B).

#### aSNR/aNMCNR calculation

Values for aSNR and aNMCNR extracted from the STIR sequence were significantly increased at the proximal measuring point for patients reporting hypoesthesia. aSNR and aNMCNR showed significantly higher values in these patients compared to the contralateral side (aNMCRN: 5.921 vs. 2.895 a.u., p = 0.007, aSNR: 10.62 vs. 7.011 a.u., p = 0.040) and to healthy controls (5.921 vs. 3.103 a.u., p = 0.021) (Table 2). Good intrareader correlations could be calculated for the aNMCNR and the aSNR proximally (ICC = 0.797, p < 0.001 and ICC = 0.690, p = 0.002). Additionally, a significant difference was revealed for patients with neurosensory impairments compared to symptom-free patients (5.921 vs. 2.956 a.u., p = 0.040) (Fig. 1C,D).

**Table 2:** This table shows the median signal-to-noise ratios (aSNR and aNMCNR for the STIR sequence) for fracture side in a proximal and distal location for the IAN in patients suffering a neurosensory deficit.

	aNMCNR		aSNR	
	Contralateral	Healthy controls	Contralateral	Healthy controls
Proximal (n = 8)	5.921 vs. 2.895, p = 0.007	5.921 vs. 3.103, p = 0.021	10.62 vs. 7.011, p = 0.040	10.62 vs. 8.864, p = 0.160
Distal (n = 8)	2.965 vs. 2.895, p = 0.712	2.965 vs. 3.103, p = 0.214	9.578 vs. 7.011, p = 0.547	9.578 vs. 8.864, p = 0.945

Significant differences could be detected for the IAN in terms of increased aSNR and aNMCNR proximal of the fracture location compared to the contralateral side and to healthy controls.

**Figure 2:**
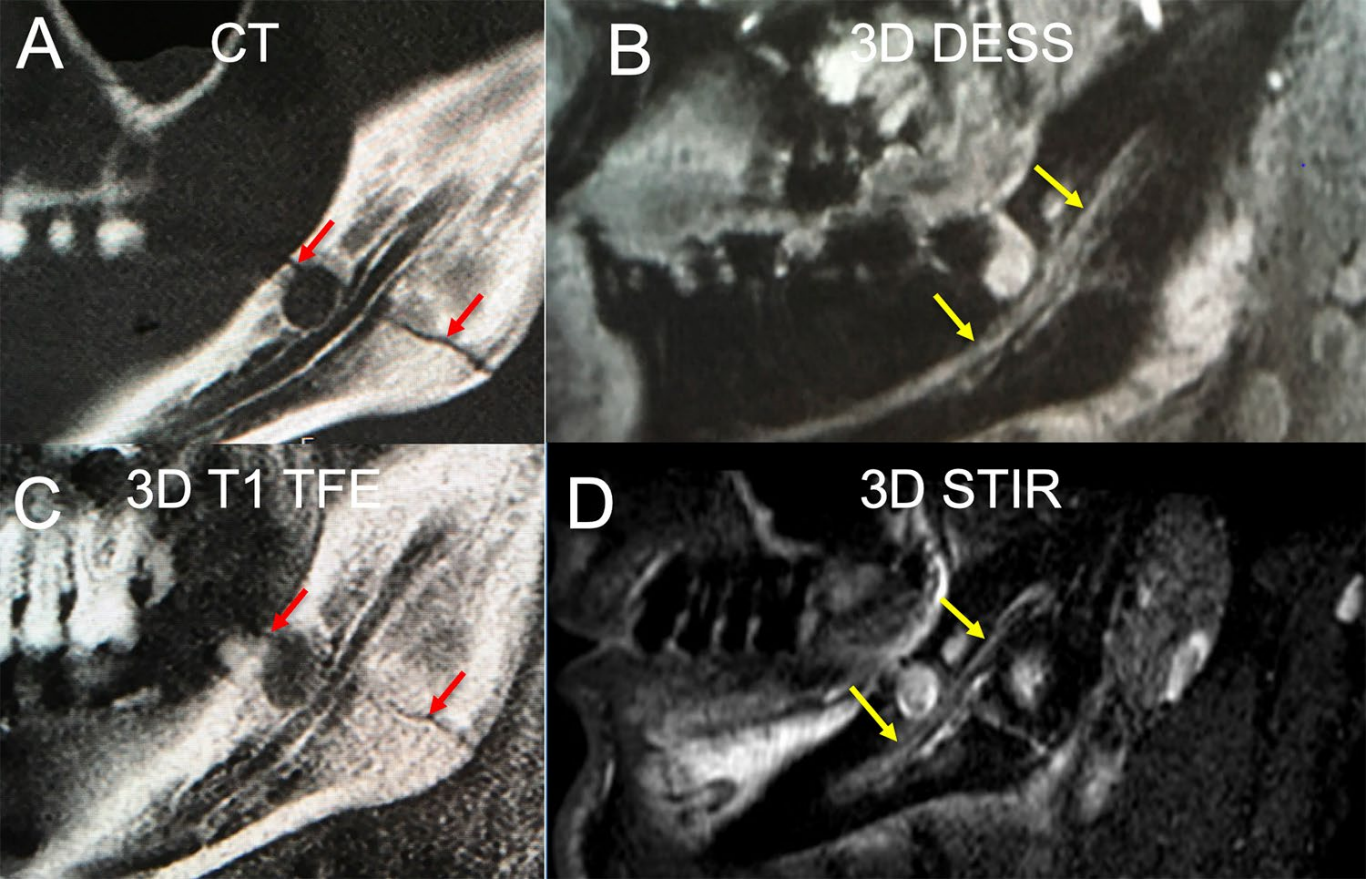
In this clinical case a slightly displaced mandible fracture is illustrated (red arrows). The CT (**A**) and the 3D T1 FFE sequence (**C**) revealed the osseous continuity disruption with involvement of the mandibular canal with comparable accuracy. However, no conclusions regarding the condition of the IAN can be drawn as it is not visualized sufficiently. The DESS sequence allows for precise depiction of the IAN (**B**). The STIR sequence shows an increase in signal intensity within the IAN as well as an enlarged nerve diameter (**D**). The nerve continuity is preserved (yellow arrows).

**Figure 3:**
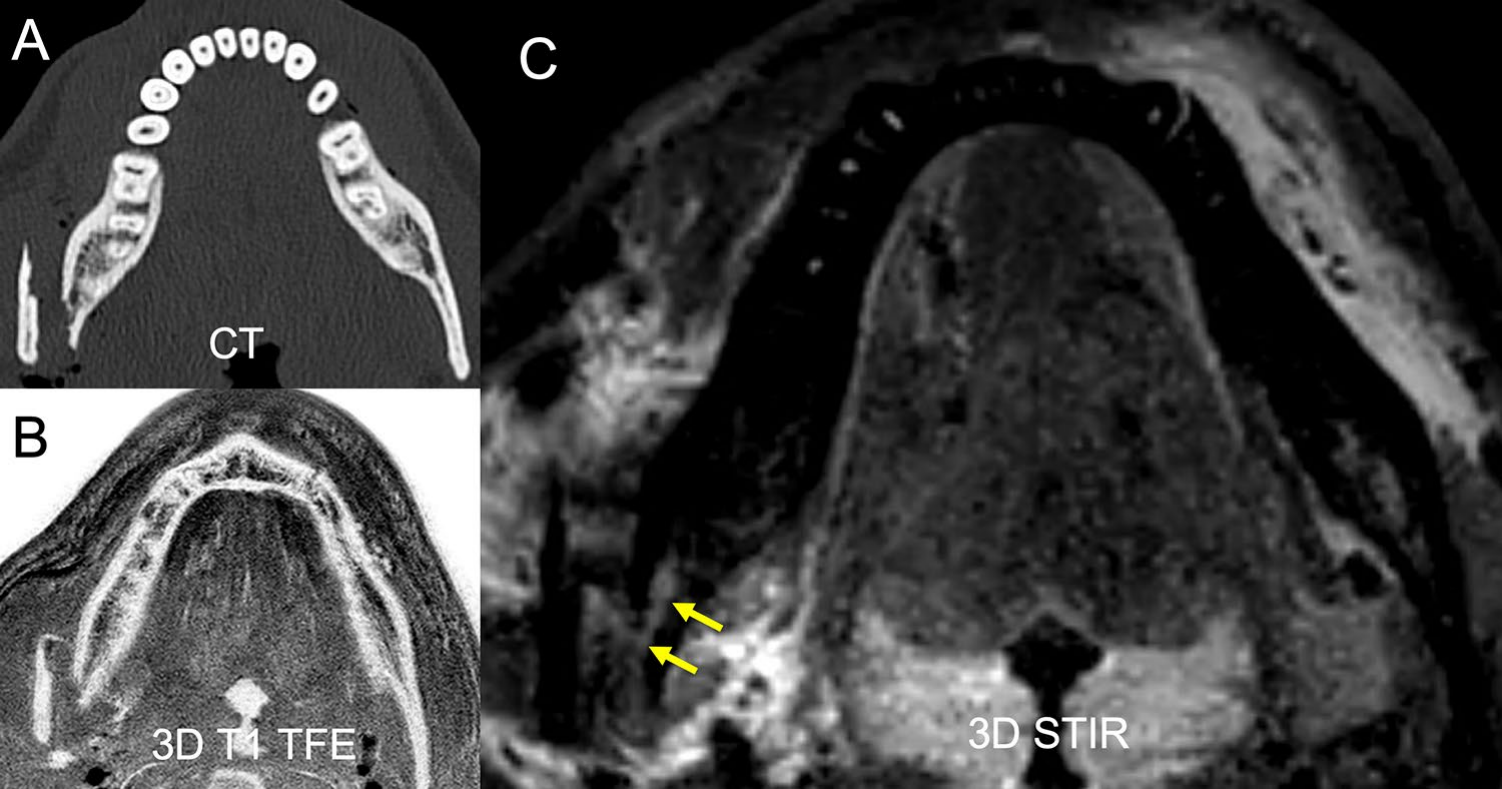
In this clinical case a highly displaced mandible fracture is illustrated. The CT (**A**) and the 3D T1 FFE sequence (**B**) revealed the osseous continuity disruption with involvement of the mandible canal with comparable accuracy. However, no conclusions regarding the condition of the IAN can be drawn as it is not visualized sufficiently. The STIR sequence shows an increase in signal intensity within the IAN as well as an enlarged nerve diameter (**C**). The nerve continuity is preserved (yellow arrows).

In distal measurements as well, a tendency towards higher values in symptomatic patients was detected, a level of significance was not reached (2.965 vs. 1.259 a.u, p = 0.099). Figures 2 and 3 depict fracture constellations with IAN insults visualized using the presented protocol. The DESS sequence did not provide significant results and could be eliminated from the protocol before clinical implementation to reduce scan time.

## Discussion

This feasibility study presents an MRI protocol for rapid imaging and assessment of IAN damage in trauma settings. The image quality and the diagnostic accuracy of all three sequences (3D STIR, 3D T1 FFE and 3D DESS sequence) was assessed by two different radiologists as “very good” and was only diminished by metal artifacts in the course of fractures. The fracture location could be precisely depicted using the 3D T1 FFE sequence, and had comparable displacement values to CT. Additionally, the 3D STIR sequence, a sequence highly sensitive to edema formation in various tissue entities, allowed for direct visualization of the IAN course, and displayed significantly higher aSNR and aNMCNR values for patients with neurosensory deficits. Using aSNR and aNMCNR allowed for calibration of the nerve signal alterations with regard to the standard deviation (aSNR) and in comparison to the surrounding muscle tissue (aNMCNR). This approach enabled the clinician for signal quantification. An enlarged nerve diameter proximal to the fracture location was observed when compared to the healthy contralateral sides, healthy controls, and patients reporting no symptoms of nerve function impairment. The increased diameter may represent neuronal swelling, a possible initial hallmark of Wallerian Degeneration. In comparison, fracture dislocation using CT was not associated with the presence of hypoesthesia in this study, which however could be due to the low cohort size.

The data acquired in this study are in accordance with previous studies on structural features of the IAN, and have a high reproducibility in displaying nerve morphology and structural features^21,22^. Agbaje et al. displayed the inferiority of 3D CT tracings to determine IAN location and diameter when compared to MRI in cadavers^23^. In cases of highly atrophied mandibles, CBCT may also be unable to distinguish close anatomic structures. These facts point to the modality-specific inherent limits. The presented MRI protocol allowed for the visualization of osseous structures at comparable levels to CT. Additionally, the MRI was able to depict hallmarks of nerve damage such as edema, involvement of nerve sheaths and fascicular structures, internal neuroma formation or Wallerian degeneration.

Several groups have tried to establish an association between imaging and histology based on the Sunderland classification of nerve injuries^10,11,22^. According to Sunderland, there are 5 histologic grades of nerve injury. These classifications can be used as prognostic indicators for spontaneous nerve recovery. Surgical intervention is indicated in Sunderland Grade IV and V, and may be indicated in Grade III^24^. The only suitable correlate for injury patterns of Sunderland Grade I–IV is quantification of differences in nerve thicknesses and nerve edema, presumably indicating presence and degree of nerve damage.

As one amongst few previous attempts of visualizing damage to IAN Kress et al. used a MRI protocol comprising a proton density weighed sequence as well as T1-weighed contrast enhanced sequence^25^. Kress et al. showed that it is possible to visualize the IAN in 21 out of 23 patients suffering from a fracture of the mandible angle. Defects in nerve continuity could be shown and percent differences in signal intensity could be determined between fracture patients and healthy cohorts after contrast agent administration. However, no differences could be detected between patients with neurosensory deficits when compared to symptom-free controls.

In our feasibility study, MRI without contrast agent was performed to visualize osseous structures and soft tissues to determine feasibility of a rapid assessment in an emergency setting. MRI, unlike CT and CBCT, can directly assess neurovascular structures. Direct examination of the nerve allows for visualization of injury site and extent, determining the need for surgical repair and guiding microneurosurgical reconstruction in rare cases of neurotmesis.

When interpreting the results of the conducted study a number of methodical limitations have to be considered. The first limitation of any feasibility study lies in a small cohort size, and in this study, a narrow distribution of age in both the patients and healthy volunteers. Future research can study more advanced age groups, as well as anatomic peculiarities like edentulous and atrophied mandibles. Second, all MR imaging was performed immediately after traumatic insult, and edema and hematoma formation may negatively affected image quality due to signal alterations in the adjacent soft tissues like in the musculature. Third, varying MRI protocols and sequences on different scanners need subsequent testing to replicate results and ensure adequate diagnostic quality can be achieved with different technical equipment. Prior to this, the application of the described imaging protocol with the dedicated sequence parameters was necessary to achieve comparable imaging quality. Fourth, in subjects with implants, metallic restorations or osteosynthesis material, artifacts may reduce the image quality. Reducing this artifact is actively being investigated^26^. However, current limitations may hamper the ability to monitor nerve signal alterations in the long term and compare them to the baseline values. This might complicate the establishment of quantitative predictive values in the future. The goal of future studies might be to investigate on the prospective value of detected signal alterations of the IAN due to mandible fractures or during the consecutive treatment in clinical and imaging follow-up examinations to elaborate the clinical significance of the presented results.

## Conclusion

The presented prospective study substantiates the role of MRI in mandible fractures with suspected nerve involvement. The described imaging protocol provides high resolution sequences allowing for the precise, direct visualization of the IAN neurovascular bundle from the foramen ovale to the foramen mentale. These imaging findings were compared to clinical symptoms. In patients with hypoesthesia following mandible fracture, increased aNMCNR, aSNR and nerve diameter on MR imaging may give evidence or an indication for concurrent nerve repair at time of surgery.

## Abbreviations

aNMCNR: Apparent nerve-muscle contrast-to-noise ratio
aSNR: Apparent signal-to-noise ratio
CBCT: Cone beam computed tomography
CI: Confidence interval
CT: Computed tomography
CNR: Contrast-to-noise ratio
DESS: Double echo steady state
IAN: Inferior alveolar nerve
MDCT: Multi-detector computed tomography
MRI: Magnetic resonance imaging
ROI: Region of interest
SD: Standard deviation
SI: Signal intensity
STIR: Short tau inversion recovery
